# Supplementary material for: Defective transcription of AAGAG satellite DNA causes sex-ratio meiotic drive in Drosophila
Source: Nat Commun. 2026 Apr 25;17:5701. doi: 10.1038/s41467-026-72480-y (PMC13320161; doi:10.1038/s41467-026-72480-y)
Supplement: Supplementary file 2 — Reporting Summary [file 41467_2026_72480_MOESM2_ESM.pdf]

nature portfolio

Corresponding author(s): Yukiko Yamashita

Last updated by author(s): Apr 4, 2025

Reporting Summary

Nature Portfolio wishes to improve the reproducibility of the work that we publish. This form provides structure for consistency and transparency in reporting. For further information on Nature Portfolio policies, see our Editorial Policies and the Editorial Policy Checklist.

Statistics

For all statistical analyses, confirm that the following items are present in the figure legend, table legend, main text, or Methods section.

n/a	Confirmed	
☐	☒	The exact sample size (*n*) for each experimental group/condition, given as a discrete number and unit of measurement
☐	☒	A statement on whether measurements were taken from distinct samples or whether the same sample was measured repeatedly
☐	☒	The statistical test(s) used AND whether they are one- or two-sided<br>*Only common tests should be described solely by name; describe more complex techniques in the Methods section.*
☒	☐	A description of all covariates tested
☒	☐	A description of any assumptions or corrections, such as tests of normality and adjustment for multiple comparisons
☐	☒	A full description of the statistical parameters including central tendency (e.g. means) or other basic estimates (e.g. regression coefficient) AND variation (e.g. standard deviation) or associated estimates of uncertainty (e.g. confidence intervals)
☐	☒	For null hypothesis testing, the test statistic (e.g. *F*, *t*, *r*) with confidence intervals, effect sizes, degrees of freedom and *P* value noted<br>*Give P values as exact values whenever suitable.*
☒	☐	For Bayesian analysis, information on the choice of priors and Markov chain Monte Carlo settings
☒	☐	For hierarchical and complex designs, identification of the appropriate level for tests and full reporting of outcomes
☒	☐	Estimates of effect sizes (e.g. Cohen's *d*, Pearson's *r*), indicating how they were calculated

Our web collection on statistics for biologists contains articles on many of the points above.

Software and code

Policy information about availability of computer code

Data collection	This study analyzed publicly available data from Raz et al., 2023; Gartner et al., 2019; modENCODE 2010.
Data analysis	FindMarkers tool in Seurat v5.1

For manuscripts utilizing custom algorithms or software that are central to the research but not yet described in published literature, software must be made available to editors and reviewers. We strongly encourage code deposition in a community repository (e.g. GitHub). See the Nature Portfolio guidelines for submitting code & software for further information.

Data

Policy information about availability of data

All manuscripts must include a data availability statement. This statement should provide the following information, where applicable:

- Accession codes, unique identifiers, or web links for publicly available datasets
- A description of any restrictions on data availability
- For clinical datasets or third party data, please ensure that the statement adheres to our policy

All study data are included in the article.

Research involving human participants, their data, or biological material

Policy information about studies with human participants or human data. See also policy information about sex, gender (identity/presentation), and sexual orientation and race, ethnicity and racism.

Reporting on sex and gender	N/A
Reporting on race, ethnicity, or other socially relevant groupings	N/A
Population characteristics	N/A
Recruitment	N/A
Ethics oversight	N/A

Note that full information on the approval of the study protocol must also be provided in the manuscript.

Field-specific reporting

Please select the one below that is the best fit for your research. If you are not sure, read the appropriate sections before making your selection.

☒ Life sciences ☐ Behavioural & social sciences ☐ Ecological, evolutionary & environmental sciences

For a reference copy of the document with all sections, see nature.com/documents/nr-reporting-summary-flat.pdf

Life sciences study design

All studies must disclose on these points even when the disclosure is negative.

Sample size	No statistical method was used to determine the sample size. For all biological samples, the maximum possible sample size was chosen for each type of data.
Data exclusions	No data were excluded from the analyses.
Replication	All data were collected during independent trials conducted on separate days. All attempts at replication were successful.
Randomization	Randomization is not relevant to our study, because wild-type control data were compared to mutant data.
Blinding	Blinding is not relevant to our study, because during analyses wild-type control and mutant data sets are easily identified. Blinding was not performed during data acquisition and/or analysis.

Reporting for specific materials, systems and methods

We require information from authors about some types of materials, experimental systems and methods used in many studies. Here, indicate whether each material, system or method listed is relevant to your study. If you are not sure if a list item applies to your research, read the appropriate section before selecting a response.

Materials & experimental systems

n/a	Involved in the study	
☐	☒	Antibodies
☒	☐	Eukaryotic cell lines
☒	☐	Palaeontology and archaeology
☐	☒	Animals and other organisms
☒	☐	Clinical data
☒	☐	Dual use research of concern
☒	☐	Plants

Methods

n/a	Involved in the study	
☒	☐	ChIP-seq
☒	☐	Flow cytometry
☒	☐	MRI-based neuroimaging

Antibodies

Antibodies used	The primary antibodies used were anti-Mst77F (1:1000 dilution; guinea pig (Park et al., 2023)) and anti-H3K9me3 (1:200 dilution; rabbit; Abcam ab8898). The secondary antibodies used were anti-rabbit IgG conjugated with Alexa Fluor 647 (1:200 dilution; goat;

	Abcam ab150079) and anti-guinea pig IgG conjugated with Alexa Fluor 568 (1:200 dilution; goat; Abcam ab175714).
Validation	The anti-Mst77F was validated by Park et al., 2023. All other antibodies were validated by the manufacturer. Antibodies obtained from commercial vendors or colleagues were tested by immunofluorescence analyses to ensure that they recognize the correct antigen.

Animals and other research organisms

Policy information about studies involving animals; ARRIVE guidelines recommended for reporting animal research, and Sex and Gender in Research

Laboratory animals	Drosophila malanogaster y1w1 (BDSC 1495), nos-GAL4:VP16(Van Doren et al., 1998), bam-GAL4:VP16 (Chen and McKearin, 2003), Ubi-GFPS65C:α1-tub84B (Rebollo et al., 2004), H3.3A-Dendra2 (Shindo and Amodeo, 2019), C(1)RM/C(1;Y)6, y1w1f1/O (BDSC 9460), UAS-TRiP.HMS01699 (BDSC 38255; UAS-HP2RNAi), UAS- AAGAGshRNA (gift from Gary Karpen (Mills et al., 2019)), Su(var)2-HP2-GFP.FPTB (BDSC 68184; hp2-gfp), UAS-HP2-S (this study), UAS-HP2-L (this study), Ithaca I16 and Beijing B52 of Global Diversity lines (gift from Andrew Clark (Wei et al., 2014))
Wild animals	The study did not involve wild animals.
Reporting on sex	Findings specifically apply to male (sperm development).
Field-collected samples	The study did not involve field-collected samples.
Ethics oversight	Work on Drosophila melanogaster does not require ethical oversight or experimental approval.

Note that full information on the approval of the study protocol must also be provided in the manuscript.

Plants

Seed stocks	N/A
Novel plant genotypes	N/A
Authentication	N/A
