# Supplementary material for: Defective transcription of AAGAG satellite DNA causes sex-ratio meiotic drive in Drosophila
Source: Nat Commun. 2026 Apr 25;17:5701. doi: 10.1038/s41467-026-72480-y (PMC13320161; doi:10.1038/s41467-026-72480-y)
Supplement: Supplementary file 3 — Transparent Peer Review File [file 41467_2026_72480_MOESM3_ESM.pdf]

nature portfolio

Peer Review File

Defective transcription of AAGAG satellite DNA causes sex-ratio meiotic drive in Drosophila

Corresponding Author: Professor Yukiko Yamashita

This file contains all reviewer reports in order by version, followed by all author rebuttals in order by version.

Version 0:

Reviewer comments:

Reviewer #1

(Remarks to the Author)
This manuscript by Kumon, Yamashita, and colleagues presents two major claims:

1) Transcription at the AAGAG satellite DNA, driven by HP2, is required for proper histone-to-protamine exchange during spermiogenesis:
The authors argue that it is the act of transcription - rather than the presence of repeat-derived transcripts - is critical for this process. This conclusion is based on comparisons between HP2 knockdown (KD) and AAGAG repeat RNA KD. Both KDs led to a near-complete loss of AAGAG RNA. However, in the AAGAG KD, antisense CUCUU transcripts remained unaffected, and protamine incorporation proceeded normally, whereas in HP2 KD, CUCUU transcription was reduced, and protamine incorporation defects were observed. The authors propose that transcription at the repeat loci primes the chromatin for histone-to-protamine exchange, explaining why HP2 KD - but not AAGAG KD - disrupts protamine incorporation. They explained that infertility observed in AAGAG KD flies in their and Karpen group's experiments (Mills, 2019, PMID: 31687931) may be due to KD of AAGAG-containing Y-linked fertility genes, not KD of AAGAG RNA itself.

2) HP2 acts as a suppressor of sex-ratio meiotic drive:
In HP2 KD flies, Y-chromosome-bearing spermatids, which have more abundant AAGAG satellite repeats, are more prone to defects in histone-to-protamine exchange. The authors demonstrate that reducing AAGAG satellite DNA content (via a naturally occurring chromosome II variant with lower AAGAG repeat content) can rescue these defects.

The manuscript poses an intriguing question about the role of pervasive transcription in the testis and proposes an interesting model linking transcription at satellite DNA to chromatin reorganization and sex-ratio distortion. The writing is clear, the data are presented well, and I appreciate the attention given to parts of the methods section.

However, I find that the data currently provided are not sufficient to substantiate the authors' claims with the level of confidence expressed in the manuscript. I recommend that the authors either provide additional supporting data or temper their conclusions accordingly.

Major Points:
1) HP2 as a transcriptional activator:
The authors imply that HP2 directly activates AAGAG repeat transcription, but the evidence is weak and indirect effects cannot be ruled out. In Supplementary Fig. 2C, AAGAG transcription appears to anti-correlate with HP2-GFP expression, which is difficult to reconcile with a model where HP2 acts as a direct activator. Is this image representative?
Moreover, the ChIP-seq data show only modest AAGAG enrichment. The comparison should be between HP2 enriched DNA and a mock ChIP control, rather than genomic abundance, given ChIP and sequencing biases.
Without genome-wide binding or transcriptional profiling, it remains unclear whether AAGAG is a primary target or one of many weak targets of HP2, especially given HP2’s known association with HP1, broad distribution on the polytene chromosome, and its role in transcriptional silencing (Shaffer, 2006, PMID: 16980400).
I recognize that testing binding at repeats is technically challenging. However, could the authors provide cytological evidence? For instance, if they cross in HP2-GFP in XO male or the male with II[delta AAGAG], do they see any HP2-GFP distribution differences in spermatocytes where each chromosome has distinct territories?
If no additional evidence can be provided, the authors should revise their conclusions to state that HP2 is required for

AAGAG transcription, but not necessarily a direct activator (e.g. lines 113-114).

2) The act of transcription versus the transcripts:
The authors propose that transcription itself modifies chromatin to facilitate histone-to-protamine exchange. This is plausible, but the current data do not directly demonstrate that chromatin changes occur at the repeat locus or that HP2 KD affects only AAGAG transcription. HP2 KD may influence expression of many other genes involved in chromatin remodeling or protamine incorporation. As the authors themselves acknowledged the opposing view in their introduction that transcription could be a consequence of open chromatin rather than a driver, stronger evidence is needed to support their model.
Could the authors transgenically express AAGAG (Mills, 2019, PMID: 31687931) in their HP2 KD to show that AAGAG RNA does not rescue protamine incorporation defect?
Could the authors assess chromatin state (e.g., H3K9me3 ChIP) at AAGAG repeats in wild-type versus HP2 KD testes? ChIP is not a requirement; any way to substantiate their claim in an orthogonal way would suffice.

3) Total AAGAG amount correlates with sperm DNA compaction defects in HP2 KD:
The data suggesting that total AAGAG levels influence sperm chromatin compaction are interesting, with implications for sex-ratio meiotic drive. However, the mechanistic model is unclear: how does deletion of AAGAG on chromosome II in trans rescue defects arising from AAGAG on the Y chromosome?
Do the authors observe physical clustering of AAGAG repeats across chromosomes, as described for other repeats (Jagannathan and Yamashita papers)?
Moreover, the II[ΔAAGAG] chromosome is a natural variant and likely harbors many other genetic differences. Thus, it is difficult to attribute the rescue solely to reduced AAGAG levels.
Are there alternative approaches or independent reagents the authors could use to manipulate AAGAG abundance more specifically? For example, in XO males (which lack a Y chromosome and have reduced AAGAG content), does protamine exchange proceed normally with HP2 KD?

Minor Point - RNAi rescue controls:
I am concerned that using BamGal4; UAS-HP2-RNAi rescued by BamGal4; UAS-HP2-RNAi, UAS-HP2(L/S) could artificially weaken RNAi efficiency due to the titration of Gal4 across two UAS constructs. Can the authors include an additional control, such as BamGal4; UAS-HP2-RNAi + UAS-XXX(e.g. GFP), to verify that RNAi remains effective with two UAS elements?
Even better, are there any hypomorphic HP2 mutants they could use from Elgin studies?

Overall, the manuscript presents a very interesting story that would be a valuable contribution to the field. However, additional data or revisions to the interpretation are needed to match the strength of the claims made.

Reviewer #2

(Remarks to the Author)
In this paper, the authors demonstrate that the heterochromatin protein HP2 is important for transcribing the abundant simple satellite AAGAG in Drosophila melanogaster spermatocytes. Their results show that disrupting HP2 leads to spermatid chromatin abnormalities and this phenotype correlates with the abundance of the AAGAG satellite. They propose an interesting new model suggesting that satellite transcription in spermatocytes is important for remodeling chromatin and further speculate that this process may be involved in meiotic drive.

While overall interesting and potentially important for the field, there are some major issues that should be addressed relating to the methods, text, and representation of the results. The conclusions about meiotic drive are speculative as presented, and the paper title should be revised. See below.

• There is no quantitative analysis to accompany Figure 1 in the supplement and there are insufficient details in the materials and methods to assess the single cell and proteomic analysis.

• Figure 2: It is unclear how the measurements in panel b were made. Please include more information about how the images were analyzed both in the legend and in the methods. Is each dot global intensity for each testis? Per cyst? Where is the variance?

What is the x-axis in Figure 2C? How is abundance in the genome quantified? What conclusions can be confidently draw from these analyses? Showing the abundance of each satellite in the ChIP input would be more convincing to demonstrate that the apparent enrichment is unrelated to its sheer abundance in the genome. Quantitative comparison of simple tandem repeats in Illumina data is error-prone.

• Figure 3: Fly age might contribute to the phenotype. Can you report the ages of the flies for these experiments? In Fig 3C+D, what is the rationale for not using primers that span exon-exon junctions for all genes?

• Figure 4: The barplot is confusing and seems to be at odds with the cytological data. Based on the representative image shown in 4C, only a small subset of spermatids in the bundle have H3K9me3 staining. What is the difference between the images in c and d? The barplot cannot represent the fraction of H3K9me3 spermatids in a bundle based on the images.

Does this represent the fraction of bundles with H3K9me3 positive in any spermatids, or any needle-shaped spermatids, and why do you make this distinction? This plot could be misleading. Also, the plot style is difficult to interpret. If you keep with this mode of plotting, then include a key with colors instead of writing the text in the plot.

The spermatids may be failing properly complete the H2P transition, but it is unclear how specific this defect is to H3K9me3 retention compared to other histone marks. I suggest softening the language (e.g. line 194-196) to better reflect your results. Also, please add statistics to your plots.

• Figure S4: Similar to figure 4, it is hard to decipher the bar plot. These two bars are not comparable, and the presentation is confusing. How do you define defective spermatids? Defining based on the lack of Mst77F staining would be tautological (i.e.,100% of defective spermatids are Mst77F negative). Please try plotting a different way and provide statistics in the plots. It would also be helpful to stain for other components of mature sperm chromatin like protamines.

• Figure 5: Please show more representative images to support your claim. This figure shows equal representation of defective X and Y-spermatids, not an overrepresentation of defective Y spermatids.

The barplot with percentage in 5B does not seem like the best way to present those data. Consider using something where we can see the variance. For figure 5A, how many individuals do you use in these crosses? I don't see these crosses described in the methods, this needs to be added. Please also consider adding a supplemental table that includes the total number of progeny counted for each replicate. If the progeny counts are representative of those in the HP2RNAi part of figure 3A (<60 offspring, and generally <<60), then I am not sure how confident you can be that you are observing sex ratio distortion due to meiotic drive. Additionally, please reconsider the statistical tests you use to contrast your data. What are you comparing? The two genotypes or if the two genotypes are different from 50%?

• Section titled “HP2 depletion preferentially harms Y-chromosome-containing spermatids, leading to sex ratio meiotic drive”: Given the abundance and widespread nature of AAGAG satellite, the distortion in sex ratio is surprising. The 2nd chromosome has nearly as much AAGAG as the Y chromosome. The distortions reported in Figure 6 are so slight for having such a large difference in abundance (there is a similarly large difference in abundance between the X and Y). Therefore, the model proposed in this paper doesn't seem to be strongly supported by the data and the language should be tempered. Additionally, please choose plots and summaries that show all of your data in figure 6D, rather than these barplots.

There is no difference in representation of 2nd chromosomes with and without the deletion in AAGAG in female offspring, suggesting that there might be something else happening. Alternative hypotheses should be presented. How are you controlling for viability effects of the AAGAG-deletion sex chromosomes, which might also explain some of your data? Do you see mendelian segregation of the 2nd chromosomes through female meiosis?

I'm also concerned about the data in fig 6b showing that heterozygotes have such a strong reduction in defects. Your y axis here suggests that you are counting abnormal sperm per cyst whereas elsewhere it seemed like you were counting cysts with defective sperm. The data should be consistently evaluated across all figures. It would be nice to see both representations: number of cysts with issues and number of defective sperm in cysts, although the latter should be difficult to quantify. Consider also averaging per testis.

How did you analyze the 3D cysts to count the defective sperm? There is some general defect in these spermatids, but it seems to be in a minority of spermatids. Given the widespread distribution of the satellite, how do you explain your cytological data? Please also reorder the panels so that the Fig 6a,b,c,d are in the same order as the other figures.

• For the evidence that supports your model: e.g. that AAGAG deletion rescues HP2 depletion phenotype, you show this in a heterozygote where the other half of spermatids with AAGAG on chromosome II, along with the other AAGAG in the genome, could show an effect but don't. To me, these results do not strongly support your hypothesis and require further explanation.

• Figure S8: There is a difference in AAGAG signal outside of chromosome II that seems to be more consistent with FISH efficiency/image quality than a deletion of AAGAG on chromosome II. A positive control will help with the interpretation. Given your claim that the Y chromosome is highly different in AAGAG abundance, you should show FISH on male brains. This is important not just for supporting your hypothesis but to show that the fly genotypes that you are looking at have stark differences in AAGAG abundance on the Y chromosome.

• The model about the role of satellite transcription and chromatin remodeling in testes is interesting and will be important for the field, but requires further testing, as transcription and important aspects of the histone-to-protamine transition were not measured directly. Measures of nascent RNA levels and PolII transcription could support this model. The further speculation about meiotic drive proposed here (in supplemental figure 9) depends on the model of satellite transcription that they proposed. I like the idea but feel that this model for meiotic drive is too speculative to center in this research paper. The paper title " HP2 prevents sex-ratio meiotic drive through transcription of AAGAG satellite DNA" should be changed to reflect the conclusions that are strongly supported by the results. The current title makes it sound like the role of HP2 is to prevent meiotic drive.

Minor comments:
• Figure 1: Please add percentages to your pie charts

• Lines 106-107: I was confused by the 2nd half of this sentence "with more severe reduction of AAGAG RNA", as it does not

seem to follow the first half.

• Supplemental Figure 2: Add quantifications for panel f.

• Supplemental Figure 7: It was difficult to figure out the colors.

Reviewer #3

(Remarks to the Author)
This is an interesting manuscript that provides new information about the role of transcription of satellite DNAs, and discovers a new function for the HP2 protein. The results are largely straightforward and of substantial interest to research in gamete biology and meiotic drive field.

One question is how the RNA interference is working. RNAi is supposedly cytoplasmic but here it appears to be affecting transcripts in the nucleus. How so?

The manuscript is generally clearly written.

Other points:

Fig 2C. Are the satellite abundance estimates mean values across populations?

Supplementary figures and legends have quite a few minor grammar errors and odd usage of English.

Version 1:

Reviewer comments:

Reviewer #1

(Remarks to the Author)
I recommend publication of the revised manuscript, pending two minor clarifications detailed below.

I appreciate the authors' additional experiments using an independent AAGAG deletion line to assess sperm differentiation and HP2 localization. These data strengthen the conclusions regarding HP2 action on AAGAG DNA and the dose-dependent effect of AAGAG on sperm DNA condensation. While more experiments are needed to obtain definitive answers to whether HP2 functions as a direct activator and whether a formal checkpoint exists, I believe the authors did due diligence in their experiments and worded these interpretations as speculative.

Minor points:

Lines 112–113:
"However, given that HP2 expression decreased at the time of strongest AAGAG/CUCUU transcription in spermatocytes (Fig. 1g) …"
Is Fig. 1g the best figure for this sentence? Fig. S4a shows HP2 expression, but not Fig 1g.

Lines 188–190:
"Similar to the retention of H3K9me3, H3.3 histone was also retained in the defective spermatids in bam>HP2RNAi, whereas another histone mark, AcH4 …"
I could not find the AcH4 data referenced here.

Reviewer #2

(Remarks to the Author)
I appreciate the clarifications provided in the response and the paper.

Regarding the response about the original figure S8: I can appreciate the point about sample-to-sample signal variance but I would hope that this approach would work for detecting large differences, especially with a control. The published sequence-based estimates might not represent the flies in your hands, especially in repeat regions. I recommend going back to the original presentation and showing more images than the one, ideally from males, and then present the published genomic estimates alongside these images.

Reviewer #3

(Remarks to the Author)
The authors have addressed my previous concerns.

Version 2:

Reviewer comments:

Reviewer #1

(Remarks to the Author)
The authors fixed minor errors, and I recommend the manuscript for publication.

Reviewer #2

(Remarks to the Author)
Thank you for your response, I am satisfied with the revised figure. Congratulations on this interesting work.

Open Access This Peer Review File is licensed under a Creative Commons Attribution 4.0 International License, which permits use, sharing, adaptation, distribution and reproduction in any medium or format, as long as you give appropriate credit to the original author(s) and the source, provide a link to the Creative Commons license, and indicate if changes were made.
In cases where reviewers are anonymous, credit should be given to 'Anonymous Referee' and the source.
The images or other third party material in this Peer Review File are included in the article's Creative Commons license, unless indicated otherwise in a credit line to the material. If material is not included in the article's Creative Commons license and your intended use is not permitted by statutory regulation or exceeds the permitted use, you will need to obtain permission directly from the copyright holder.
To view a copy of this license, visit https://creativecommons.org/licenses/by/4.0/

Point-by-point responses:

Reviewer comments are in blue

Reviewer #1 (Remarks to the Author):

This manuscript by Kumon, Yamashita, and colleagues presents two major claims:

1) Transcription at the AAGAG satellite DNA, driven by HP2, is required for proper histone-to-protamine exchange during spermiogenesis:
The authors argue that it is the act of transcription - rather than the presence of repeat-derived transcripts - is critical for this process. This conclusion is based on comparisons between HP2 knockdown (KD) and AAGAG repeat RNA KD. Both KDs led to a near-complete loss of AAGAG RNA. However, in the AAGAG KD, antisense CUCUU transcripts remained unaffected, and protamine incorporation proceeded normally, whereas in HP2 KD, CUCUU transcription was reduced, and protamine incorporation defects were observed. The authors propose that transcription at the repeat loci primes the chromatin for histone-to-protamine exchange, explaining why HP2 KD - but not AAGAG KD - disrupts protamine incorporation. They explained that infertility observed in AAGAG KD flies in their and Karpen group's experiments (Mills, 2019, PMID: 31687931) may be due to KD of AAGAG-containing Y-linked fertility genes, not KD of AAGAG RNA itself.

2) HP2 acts as a suppressor of sex-ratio meiotic drive:
In HP2 KD flies, Y-chromosome-bearing spermatids, which have more abundant AAGAG satellite repeats, are more prone to defects in histone-to-protamine exchange. The authors demonstrate that reducing AAGAG satellite DNA content (via a naturally occurring chromosome II variant with lower AAGAG repeat content) can rescue these defects.

The manuscript poses an intriguing question about the role of pervasive transcription in the testis and proposes an interesting model linking transcription at satellite DNA to chromatin reorganization and sex-ratio distortion. The writing is clear, the data are presented well, and I appreciate the attention given to parts of the methods section.

However, I find that the data currently provided are not sufficient to substantiate the authors' claims with the level of confidence expressed in the manuscript. I recommend that the authors either provide additional supporting data or temper their conclusions accordingly.

We thank this reviewer for their supportive comments. Also, we are very appreciative of very important and spot-on comments by this reviewer. This reviewer suggested strengthening the three most important points either by additional experiments or textual changes. We have addressed this reviewer’s important comments, as detailed below,

Major Points:
1) HP2 as a transcriptional activator:
The authors imply that HP2 directly activates AAGAG repeat transcription, but the evidence is weak and indirect effects cannot be ruled out. In Supplementary Fig. 2C, AAGAG transcription appears to anti-correlate with HP2-GFP expression, which is difficult to reconcile with a model where HP2 acts as a direct activator. Is this image representative?
Moreover, the ChIP-seq data show only modest AAGAG enrichment. The comparison should be between HP2 enriched DNA and a mock ChIP control, rather than genomic abundance, given ChIP and sequencing biases.

Without genome-wide binding or transcriptional profiling, it remains unclear whether AAGAG is a primary target or one of many weak targets of HP2, especially given HP2's known association with HP1, broad distribution on the polytene chromosome, and its role in transcriptional silencing (Shaffer, 2006, PMID: 16980400).
I recognize that testing binding at repeats is technically challenging. However, could the authors provide cytological evidence? For instance, if they cross in HP2-GFP in XO male or the male with II[delta AAGAG], do they see any HP2-GFP distribution differences in spermatocytes where each chromosome has distinct territories?
If no additional evidence can be provided, the authors should revise their conclusions to state that HP2 is required for AAGAG transcription, but not necessarily a direct activator (e.g. lines 113-114).

This reviewer is right that we do not have the definitive evidence that HP2 is a direct regulator of AAGAG transcription. We have made this point clearer in the revised manuscript by both additional experiments and textual changes.

We are very thankful for this reviewer's understanding that biochemically profiling HP2's target in the spermatocytes is not straightforward (due to the low amount of materials available, because we would have to isolate spermatocytes only, and also simple tandem repeats are difficult to quantitatively assay, as is well-recognized in the field. We really liked this reviewer's suggestion to cytologically test the binding of HP2-GFP in various background with varying amounts of AAGAG. We conducted this experiment (shown in new Supplementary Fig S4)---which revealed that HP2 binds to chromatin in an AAGAG-dependent manner (no detectable localization was observed in delta-AAGAG strain). In AAGAG+ strain, HP2 was observed near the AAGAG, but not entirely overlapping (although due to a weak signal, we can never say HP2 is NOT there). Additionally, we agree with this reviewer that AAGAG transcription by HP2 can be indirect, thus we edited the text to reflect this point as following:

Analysis of HP2 ChIP-seq data on the modENCODE database[1] showed that HP2 binds preferentially to AAGAG satellite DNA (Fig. 2c), implying that HP2 may regulate the transcription of AAGAG satellite DNA via direct binding. Moreover, we found that HP2's localization to chromatin is dependent on AAGAG satellite DNA (Supplementary Fig. 4). However, given that HP2 expression decreased at the time of strongest AAGAG/CUCUU transcription in spermatocytes (Fig. 1g), it is possible that HP2's role in AAGAG/CUCUU transcription can be indirect. If it is direct, HP2's requirement in AAGAG/CUCUU transcription may represent an example of heterochromatin-dependent transcription[2, 3].

Related to the HP2 enrichment on AAGAG (the results presented in Fig2c), since submission, while we were analyzing another protein's enrichment on other satellite DNA, we realized that our original workflow of the analysis had a glitch. Corrected results show that AAGAG is not the top-enriched target, although still a large amount of HP2 is expected to bind to AAGAG (given that AAGAG is the most abundant sat DNA in the 'standard' Drosophila genome). Although this does not change our overall conclusion, and more importantly the results are clear that AAGAG is the only affected sat DNA upon HP2 depletion, we adjusted the text and the Figure panel. We also wanted to notify the reviewer(s) about this change, and the underlying reasoning.

2) The act of transcription versus the transcripts:
The authors propose that transcription itself modifies chromatin to facilitate histone-to-protamine exchange. This is plausible, but the current data do not directly demonstrate that chromatin

changes occur at the repeat locus or that HP2 KD affects only AAGAG transcription. HP2 KD may influence expression of many other genes involved in chromatin remodeling or protamine incorporation. As the authors themselves acknowledged the opposing view in their introduction that transcription could be a consequence of open chromatin rather than a driver, stronger evidence is needed to support their model.
Could the authors transgenically express AAGAG (Mills, 2019, PMID: 31687931) in their HP2 KD to show that AAGAG RNA does not rescue protamine incorporation defect?
Could the authors assess chromatin state (e.g., H3K9me3 ChIP) at AAGAG repeats in wild-type versus HP2 KD testes? ChIP is not a requirement; any way to substantiate their claim in an orthogonal way would suffice.

Thank you for another important comment and suggestion. We have done the experiment using UAS-AAGAG (from the Karpen lab) to test the rescue of HP2-RNAi by expression of AAGAG. Expression of AAGAG did not rescue the defects observed in HP2-RNAi (New Supplementary Figure S10), supporting our model.

Additionally, it is important to note that our results (documented in the original submission) already implied that it is likely transcription, instead of transcripts, that is important. If transcripts (AAGAG RNA) are important, then it is expected that the combination of HP2 RNAi (which reduces AAGAG RNA) and deltaAAGAG (which also reduces AAGAG RNA) should make the phenotype worse. However, in contrast to this prediction, the combination of HP2 RNAi and deltaAAGAG 'rescued' (Fig 6) HP2 RNAi phenotype, suggesting that it is not AAGAG RNA that is important. We have added this discussion more clearly in the revised manuscript. Now the discussion reads:

First, the expression of AAGAG did not rescue the bam>HP2^{RNAi} phenotype (Supplementary Figure S10), suggesting that the lack of AAGAG transcript is not the cause of the bam>HP2^{RNAi} phenotype. Furthermore, the rescue of bam>HP2^{RNAi} phenotype by introduction of chromosome II with little AAGAG satellite DNA (II$^{\Delta AAGAG}$) (Fig. 6) provides a critical support for this notion. If AAGAG RNA has an important function for spermatid development, it would be expected that deletion of AAGAG satellite DNA in II$^{\Delta AAGAG}$ worsens the phenotype of bam>HP2^{RNAi} because both conditions reduce AAGAG RNA. Instead, II$^{\Delta AAGAG}$ rescued bam>HP2^{RNAi} phenotype, implying that AAGAG RNA is dispensable for spermatid development.

3) Total AAGAG amount correlates with sperm DNA compaction defects in HP2 KD:
The data suggesting that total AAGAG levels influence sperm chromatin compaction are interesting, with implications for sex-ratio meiotic drive. However, the mechanistic model is unclear: how does deletion of AAGAG on chromosome II in trans rescue defects arising from AAGAG on the Y chromosome?
Do the authors observe physical clustering of AAGAG repeats across chromosomes, as described for other repeats (Jagannathan and Yamashita papers)?
Moreover, the II[ΔAAGAG] chromosome is a natural variant and likely harbors many other genetic differences. Thus, it is difficult to attribute the rescue solely to reduced AAGAG levels. Are there alternative approaches or independent reagents the authors could use to manipulate AAGAG abundance more specifically? For example, in XO males (which lack a Y chromosome and have reduced AAGAG content), does protamine exchange proceed normally with HP2 KD?

This paragraph by this reviewer contains two important points. First, 'why trans?':How AAGAG deletion in chromosome II can rescue the spermatid phenotype that contains Y chromosome

(with lots of AAGAG)---this ‘trans effect’ is indeed puzzling. In our original manuscript, we discussed briefly that this might reflect ‘threshold’ effect: there is a threshold on how much of DNA can be left heterochromatic (thus not incorporating protamine properly). And above this threshold level, the entire spermatid may fail to undergo sperm DNA compaction. A recent preprint from the lab of Nitin Phadnis (https://www.biorxiv.org/content/10.1101/2024.06.04.597441v1) proposes that a type of checkpoint mechanism operates in deciding whether or not to eliminate specific spermatids, which also has been repeatedly speculated [4]. We suspect that such a checkpoint might be operating in deciding whether or not to abort sperm nuclei, depending on ‘how much of DNA failed to exchange histone to protamine’. We have added a discussion, incorporating this point. Our model is explained by their concept of sperm DNA quality checkpoint, and also provides insights into the nature of this long-hypothesized checkpoint. Now the discussion reads:

It is important to note that defective spermatid development in HP2^{RNAi} is sensitive to the dose of total AAGAG carried in the haploid spermatids. We speculate that each spermatid may have a threshold of the ‘unremodeled’ chromatin it can tolerate (e.g. the amount of unremoved histones, and/or the amount of the chromatin that is not packaged by protamine), and the spermatids that have an above-threshold unremodeled chromatin may be fated for death. Indeed, the presence of a checkpoint mechanism that monitors the quality of sperm DNA has been proposed to explain the cellular phenotype of sperm-killing meiotic drivers, such as SD [4, 5]. Interestingly, the cytological phenotype of HP2^{RNAi} is reminiscent of the SD phenotype. We speculate that the AAGAG dose-sensitive nature of the HP2^{RNAi} phenotype may be due to such a checkpoint mechanism.

Related to this point, the reviewer asked whether satDNA makes cluster in the spermatid, as we reported in other cell types (early germ cells and somatic cells)---this is something we have been very curious about, however, because of highly compacted nature of spermatids, it has been difficult to obtain a satisfactory resolution to conclude whether satellite DNA clusters or not. In spermatocytes, satellite from different chromosomes do not make any cluster, as each pair of chromosomes forms ‘chromosomal territory’, separating from other pairs of homologous chromosomes.

Second, AAGAG-delta chromosome might have other mutations beyond the amount of AAGAG. Thus, it will be important to test the correlation of AAGAG and HP2-RNAi phenotype independently. To address this point, we used another AAGAG-delta strain reported by Andy Clark’s lab, and found that both delta-AAGAG strains rescues HP2RNAi phenotype (revised Figure 6). Also, it is important to note that in Fig 6, we used AAGAG-deletion strains as heterozygous, thus at least ‘recessive mutation’ causing unexpected effect is less likely. Unfortunately, XO males are sterile, failing around meiosis. Thus, we cannot use this condition to assess the relationship between AAGAG and HP2-RNAi phenotype.

Minor Point - RNAi rescue controls:
I am concerned that using BamGal4; UAS-HP2-RNAi rescued by BamGal4; UAS-HP2-RNAi, UAS-HP2(L/S) could artificially weaken RNAi efficiency due to the titration of Gal4 across two UAS constructs. Can the authors include an additional control, such as BamGal4; UAS-HP2-RNAi + UAS-XXX(e.g. GFP), to verify that RNAi remains effective with two UAS elements? Even better, are there any hypomorphic HP2 mutants they could use from Elgin studies?

Now we have added UAS-GFP as a control (new supplementary Figure S2), which showed that the addition of GFP did not rescue, suggesting that it is not weakening of the gal4 driver that led to the rescue.

Overall, the manuscript presents a very interesting story that would be a valuable contribution to the field. However, additional data or revisions to the interpretation are needed to match the strength of the claims made.

Thank you again for this encouraging comment. We hope that we addressed this reviewer's concern thoroughly.

Reviewer #2 (Remarks to the Author):

In this paper, the authors demonstrate that the heterochromatin protein HP2 is important for transcribing the abundant simple satellite AAGAG in Drosophila melanogaster spermatocytes. Their results show that disrupting HP2 leads to spermatid chromatin abnormalities and this phenotype correlates with the abundance of the AAGAG satellite. They propose an interesting new model suggesting that satellite transcription in spermatocytes is important for remodeling chromatin and further speculate that this process may be involved in meiotic drive.

While overall interesting and potentially important for the field, there are some major issues that should be addressed relating to the methods, text, and representation of the results. The conclusions about meiotic drive are speculative as presented, and the paper title should be revised. See below.

We have changed the title to more descriptive one, '**Defective transcription of AAGAG satellite DNA causes sex-ratio meiotic drive**' not to infer functionality.

• There is no quantitative analysis to accompany Figure 1 in the supplement and there are insufficient details in the materials and methods to assess the single cell and proteomic analysis.

We thank this reviewer for bringing up these important points.

We have added the images demonstrating the upregulation of AAGAG during the early spermatocyte (SC) to late SC transition (new Fig 1f) and associated quantification (Fig 1g).

We also added more details to the method about the analysis as requested.

• Figure 2: It is unclear how the measurements in panel b were made. Please include more information about how the images were analyzed both in the legend and in the methods. Is each dot global intensity for each testis? Per cyst? Where is the variance?

We have revised the method to include more details. In short, each spermatocyte (of corresponding stages) was analyzed for signal intensity (how we minimized artifactual variations is detailed in the method). Please note that during the revision, we decided that it is better to take multiple intensity measurements per nucleus and average them. Although this modified method of measurements did not change the results, because of the new method of quantification, the exact 'dots' (data points) of the graph are not identical between the original and revised submissions.

What is the x-axis in Figure 2C? How is abundance in the genome quantified? What conclusions can be confidently draw from these analyses? Showing the abundance of each satellite in the ChIP input would be more convincing to demonstrate that the apparent enrichment is unrelated to its sheer abundance in the genome. Quantitative comparison of simple tandem repeats in Illumina data is error-prone.

As we mentioned in our response to rev #1, in the course of revision, we realized that our original analysis pipeline included a glitch, thus we re-run the analysis. The results have changed slightly (although AAGAG's enrichment is still true, other satellite DNA was also identified as enriched. Also, now the left x-axis shows the ChIP input, and the right x-axis shows enrichment (HP2 ChIP/input) as this reviewer suggested. However, our RNA FISH results showed that only AAGAG/CTCTT RNA is impacted in HP2RNAi SC. This is possibly because the ChIP seq data is from adult heads). The x-axis 'enrichment score' is now explained in the method in more detail.

• Figure 3: Fly age might contribute to the phenotype. Can you report the ages of the flies for these experiments? In Fig 3C+D, what is the rationale for not using primers that span exon-exon junctions for all genes?

Always newly eclosed males (1-3 days old) were used (stated in the method). Although it is possible that certain aspects of phenotypes are affected by the age, HP2 depletion showed the phenotype from the early age, thus we did not pursue the potential impact of aging in this paper.

Regarding the primer design, for Fig3C, we wanted to first examine whether or not the functional mRNA was generated or not. To do so, we reasoned that the primer for the last/later exon was the best, because we recently reported that for some of gigantic genes (such as kl-3), some mutant can express early exons, but ultimately fails to produce functional mRNA because they cannot transcribe through the end of the gene (). To ensure that we do not miss such defects, we chose to assess the expression of last/late exons. However, for Fig 3D, because we suspected that AAGAG-RNAi might have diminished amount of ory due to 'RNAi-mediated cleavage' of AAGAG-containing intron, it was important to use the primers that spans the AAGAG-containing gigantic introns.

• Figure 4: The barplot is confusing and seems to be at odds with the cytological data. Based on the representative image shown in 4C, only a small subset of spermatids in the bundle have H3K9me3 staining. What is the difference between the images in c and d? The barplot cannot represent the fraction of H3K9me3 spermatids in a bundle based on the images. Does this represent the fraction of bundles with H3K9me3 positive in any spermatids, or any needle-shaped spermatids, and why do you make this distinction? This plot could be misleading. Also, the plot style is difficult to interpret. If you keep with this mode of plotting, then include a key with colors instead of writing the text in the plot.

We apologize for the confusion. We recognize that the x-axis labelling for this type of graphs throughout the original manuscript was confusingly worded, leading to the impression by this reviewer that the graphs do not accurately represent the data. Now we replaced the x-axis labeling and also provided the reasoning behind the method of scoring (we did not choose the method of scoring in an inconsistent manner; instead, we had to choose the method based on how best to describe the defects in the most 'fair' manner (least room for bias).

For Fig 4d, we switched the graph to a dot plot to represent the variance. But the x-axis (now better described) means '% of cysts that contain H3K9me3 spermatids /total number of cysts in a given testis' (while focusing on needle stage only, because it is the stage that most prominently shows the defects in HP2-). We believe that this reviewer interpreted that the graph showed 'how many nuclei are defective in each given cyst', leading to their comment that the graph and the image do not match. While we understand that '% of defective nuclei/each cyst' may seem a more accurate method of scoring, it actually is not, because defective sperm nuclei start disintegrating and the visible defective spermatids at any given moment are not the total number of defective spermatids within a cyst.

The spermatids may be failing properly complete the H2P transition, but it is unclear how specific this defect is to H3K9me3 retention compared to other histone marks. I suggest softening the language (e.g. line 194-196) to better reflect your results. Also, please add statistics to your plots.

Please note that our original submission (original Fig S7, now S8) contained not only H3K9me3, but also H3.3 staining, which showed the retention in the detective nuclei (but less drastic than H3K9me3). These data indicate that indeed histone is more retained in the defective nuclei. Additionally, we also looked at another histone mark (acH4), which appears to be unaffected, although this histone mark is only present until the early canoe stage, which is before HP2^{RNAi} nuclei start exhibiting defective morphologies. We have added this to a revised Fig S7. Additionally, we adjusted the language, as we agree with this reviewer that it is too strong to make a conclusion with one retained histone marks.

• Figure S4: Similar to figure 4, it is hard to decipher the bar plot. These two bars are not comparable, and the presentation is confusing. How do you define defective spermatids? Defining based on the lack of Mst77F staining would be tautological (i.e.,100% of defective spermatids are Mst77F negative). Please try plotting a different way and provide statistics in the plots. It would also be helpful to stain for other components of mature sperm chromatin like protamines.

We have revised the Figure panel similar to the revision made to Fig 4d, as described above. We also revised the X-axis labelling (we apologize that previous labelling used a confusing language), and switched to dot plot to show the variance, with the statistics added.

• Figure 5: Please show more representative images to support your claim. This figure shows equal representation of defective X and Y-spermatids, not an overrepresentation of defective Y spermatids.

We apologize for the confusing image. We replaced the images with a more representative one. (originally, we chose the image where both X and Y defective spermatids were visible, because we previously received a review comment (for another paper) that the lack of X signal might be due to unsuccessful X FISH, and we wanted to show that the X signal is also present).

The barplot with percentage in 5B does not seem like the best way to present those data. Consider using something where we can see the variance. For figure 5A, how many individuals do you use in these crosses? I don't see these crosses described in the methods, this needs to be added. Please also consider adding a supplemental table that includes the total number of progeny counted for each replicate. If the progeny counts are representative of those in the HP2RNAi part of figure 3A (<60 offspring, and generally <<60), then I am not sure how confident you can be that you are observing sex ratio distortion due to meiotic drive.

Additionally, please reconsider the statistical tests you use to contrast your data. What are you comparing? The two genotypes or if the two genotypes are different from 50%?

We revised the method to clarify these issues. In brief, in Fig 5a, each dot is the sex ratio from individual crosses (one male x 3 wild type females).

For Fig 5b, we believe that this bar plot with Chi-square is the best way to represent the data, because each cyst does not have many defective nuclei to calculate reliable '%Y defective/total defective' for the reasons described above (each cyst may have only 3-5 defective nuclei). Please also note that we also added more numbers during the revision, thus the number is not exactly the same as our original submission.

• Section titled "HP2 depletion preferentially harms Y-chromosome-containing spermatids, leading to sex ratio meiotic drive": Given the abundance and widespread nature of AAGAG satellite, the distortion in sex ratio is surprising. The 2nd chromosome has nearly as much AAGAG as the Y chromosome. The distortions reported in Figure 6 are so slight for having such a large difference in abundance (there is a similarly large difference in abundance between the X and Y). Therefore, the model proposed in this paper doesn't seem to be strongly supported by the data and the language should be tempered. Additionally, please choose plots and summaries that show all of your data in figure 6D, rather than these barplots.

The same point was raised by reviewer #1 as well. As we discussed in the original version, which is now more elaborated, these results suggest a 'threshold' model, meaning 'total amount of unremodeled chromatin' decides whether or not the spermatid is aborted.

In the context of AAGAG+/AAGAG- second chromosome (heterozygote), we see AAGAG- chromosome survives better only if it is Y-bearing, while not much difference between AAGAG- vs. AAGAG+ if they are X bearing. The latter is not that surprising, because the earlier result of 'sex ratio' (Fig 5) suggests that X bearing sperm is surviving fairly well, even in the context of AAGAG+/AAGAG+, and not having AAGAG does not help further. However, Y bearing spermatid will be greatly helped by not having AAGAG on chromosome II. These results appear to be consistent with a type of checkpoint (akin to cells' apoptosis based on the number of DNA breaks). And a type of checkpoint consistent with our data has been long proposed (and more elaborated in a recent preprint from Nitin Phadnis' lab). We have added this discussion to the revised text.

There is no difference in representation of 2nd chromosomes with and without the deletion in AAGAG in female offspring, suggesting that there might be something else happening. Alternative hypotheses should be presented. How are you controlling for viability effects of the AAGAG-deletion sex chromosomes, which might also explain some of your data? Do you see mendelian segregation of the 2nd chromosomes through female meiosis?

As we elaborated above, X bearing spermatids are less impacted to begin with (as shown in Fig 5), and that is how we originally noticed sex ratio distortion in HP2^{RNAi} animals. If X bearing spermatids are not dying a lot to begin with, it will not 'survive more', hence no rescue by AAGAG- chromosome. We have clarified this point in the revised text.
Regarding the possibility that AAGAG deletion might have viability effects, leading to female-biased offspring: this is unlikely because AAGAG-delta stock (a natural variant isolated/characterized by Andy Clark's lab) is a healthy wild type stock, and we do not see any sex ratio in this stock.

We have not looked at AAGAG+ vs. AAGAG-delta chromosome segregation pattern (any bias or not) in female meiosis. It is an interesting possibility, and certainly deserves the attention, but none of our results and the interpretation are influenced by this possibility, we believe that this is beyond the scope of the current study.

I'm also concerned about the data in fig 6b showing that heterozygotes have such a strong reduction in defects. Your y axis here suggests that you are counting abnormal sperm per cyst whereas elsewhere it seemed like you were counting cysts with defective sperm. The data should be consistently evaluated across all figures. It would be nice to see both representations: number of cysts with issues and number of defective sperm in cysts, although the latter should be difficult to quantify. Consider also averaging per testis.

We apologize that the original version of the figures contained inconsistent method of counting. As we described above, we do not like '# of abnormal spermatid/cyst', because some abnormal spermatids disintegrate, and counting them does not accurately reflect the degree of the problem. Now we switched to a consistent method of counting and show the data as '% cyst with defective spermatids/total cyst in a testis (at needle stage)'.

Regarding the question on why heterozygous AAGAG+/- has such a strong effect---this is also the data that contributed to the threshold model (which is described in detail in our response above, to reviewer #1). We now included the checkpoint model that monitors the quality of spermatid chromatin (which is proposed by many others, and now we reference to those papers).

How did you analyze the 3D cysts to count the defective sperm? There is some general defect in these spermatids, but it seems to be in a minority of spermatids. Given the widespread distribution of the satellite, how do you explain your cytological data? Please also reorder the panels so that the Fig 6a,b,c,d are in the same order as the other figures.

To quantify defective spermatids in 3D cysts, we examined z-stacks from top to bottom for each cyst. defective spermatids, which round up, are particularly apparent at the needle stage, allowing us to reliably identify the presence of defective spermatids within a cyst. We also should note that, in spermatid stages, a cluster of spermatid nuclei that belong to a cyst are well separated from other cysts' cluster of nuclei (owing to the space occupied by elongating sperm tails). Accordingly, it is not difficult to identify the 'cluster of nuclei that belong to one cyst'.

We reordered (and renumbered) panels so that the order is not awkward.

• For the evidence that supports your model: e.g. that AAGAG deletion rescues HP2 depletion phenotype, you show this in a heterozygote where the other half of spermatids with AAGAG on chromosome II, along with the other AAGAG in the genome, could show an effect but don't. To me, these results do not strongly support your hypothesis and require further explanation.

Now we include the discussion on 'spermatid chromatin quality control checkpoint', as proposed by many researchers in the field. As a checkpoint would monitor a 'threshold level' (e.g. more than a threshold amount of DNA damage leads to cell cycle arrest), it explains well how AAGAG amount matters. We have added more detailed discussion about the sperm DNA quality checkpoint model proposed by others, and discussed our 'threshold model' in this context: more specifically, the model is that when a spermatid contains a more-than-threshold level of 'unremodelled' chromatin, it is detected by the checkpoint, and eliminated (which results in DNA

compaction defects). This would explain why the amount of AAGAG can determine whether or not the spermatid fails in DNA compaction ‘in trans’.

• Figure S8: There is a difference in AAGAG signal outside of chromosome II that seems to be more consistent with FISH efficiency/image quality than a deletion of AAGAG on chromosome II. A positive control will help with the interpretation. Given your claim that the Y chromosome is highly different in AAGAG abundance, you should show FISH on male brains. This is important not just for supporting your hypothesis but to show that the fly genotypes that you are looking at have stark differences in AAGAG abundance on the Y chromosome.

We agree with this reviewer. And there is a challenge in presenting FISH data. Although we do see signals that are consistent with delta-AAGAG strains having much less AAGAG, single images are always subject to sample-to-sample variance (e.g. some samples are stained better/worse). Therefore, we decided that it is better to provide the copy number information from the published data (the paper from Andy Clark’s lab, which quantified sat DNA copy number more quantitatively). This is shown in now Figure S9.

• The model about the role of satellite transcription and chromatin remodeling in testes is interesting and will be important for the field, but requires further testing, as transcription and important aspects of the histone-to-protamine transition were not measured directly. Measures of nascent RNA levels and PolII transcription could support this model. The further speculation about meiotic drive proposed here (in supplemental figure 9) depends on the model of satellite transcription that they proposed. I like the idea but feel that this model for meiotic drive is too speculative to center in this research paper. The paper title " HP2 prevents sex-ratio meiotic drive through transcription of AAGAG satellite DNA" should be changed to reflect the conclusions that are strongly supported by the results. The current title makes it sound like the role of HP2 is to prevent meiotic drive.

The reviewer raises important points. Importantly, however, histone-to-protamine transition cannot be the direct outcome of the transcription because transcription happens in spermatocytes, but protamine deposition occurs much later in canoe stage spermatids (more than a week later). In between these two steps, chromatin transitions from histone-based, to TP (transition protein)-based, then to protamine-based. Our proposal (since the original submission) is that the spermatocyte transcription is to replace ‘hetrochromatic’ histones with ‘non-heterochromatic’ histones, which later is important for protamine incorporation. We further clarified this point in the revised text.

Regarding the measurement of nascent RNA and pol II activity, these measurements unfortunately will not provide the specificity of satellite DNA. It is well established that spermatocytes are actively and robustly transcribing all other kinds of RNAs required for spermatid development. Indeed, we do see strong Pol II localization to the entire genome of the spermatocytes, but it does not prove that they are transcribing satellite DNA. We also have done EU incorporation, which we can see the signal, but there is no way to prove that they are satellite RNA.

We fully agree with this reviewer about the meiotic drive, and we modified the title accordingly, to “**Defective transcription of AAGAG satellite DNA causes sex-ratio meiotic drive**”. And our model on meiotic drive is only presented in the supplementary figure and the discussion, to emphasize that this is not the ‘center’ of our manuscript.

Minor comments:
• Figure 1: Please add percentages to your pie charts

We have revised the figure according to the suggestion.

• Lines 106-107: I was confused by the 2nd half of this sentence "with more severe reduction of AAGAG RNA", as it does not seem to follow the first half.

We have revised the text to clarify, but it meant that both AAGAG and CUCUU are reduced in HP2RNAi, but the reduction of AAGAG is more pronounced than the reduction of CUCUU.

• Supplemental Figure 2: Add quantifications for panel f.

We have added the quantification to the revised figure.

• Supplemental Figure 7: It was difficult to figure out the colors.

We have revised the figure according to the suggestion.

Reviewer #3 (Remarks to the Author):

This is an interesting manuscript that provides new information about the role of transcription of satellite DNAs, and discovers a new function for the HP2 protein. The results are largely straightforward and of substantial interest to research in gamete biology and meiotic drive field.

One question is how the RNA interference is working. RNAi is supposedly cytoplasmic but here it appears to be affecting transcripts in the nucleus. How so?

This reagent (AAGAG-RNAi) is described in Mills et al [6]. Indeed, the mechanism of nuclear RNAi is not well established but there are indeed cases where RNAi has been successfully utilized (ref). In this case, because AAGAG-RNAi clearly removes AAGAG-RNA based on the previous publication by the Karpen lab, we believe the use of this reagent is acceptable. However, we edited the manuscript to use more conservative language, considering the lack of full understanding concerning nuclear RNAi.

The manuscript is generally clearly written.

Other points:

Fig 2C. Are the satellite abundance estimates mean values across populations?

This was from Oregon-R-modENCODE (BDSC 25211), and is not the mean values across various populations.

On a separate note, during the revision, we noted a glitch in the workflow of our original analysis. Now the results are revised as described above in reviewer #1's comments in more detail. Also, this reviewer is right about the implication of satellite DNA abundance variations across populations. Indeed, we utilized a variant strain that contains minimal AAGAG on the 2nd

chromosome to reach the model that AAGAG amount matters in the context of HP2 RNAi. We are keen to study the impact of specific satellite DNA on various aspects of biology in the future.

Supplementary figures and legends have quite a few minor grammar errors and odd usage of English.

Thank you for pointing this out. We have gone through the supplementary figures to clean up the grammar.

References

1. mod, E.C. *et al.* Identification of functional elements and regulatory circuits by Drosophila modENCODE. *Science* **330**, 1787-1797 (2010).
2. Lu, B.Y., Emtage, P.C., Duyf, B.J., Hilliker, A.J. & Eissenberg, J.C. Heterochromatin protein 1 is required for the normal expression of two heterochromatin genes in Drosophila. *Genetics* **155**, 699-708 (2000).
3. Wei, X., Eickbush, D.G., Speece, I. & Larracuente, A.M. Heterochromatin-dependent transcription of satellite DNAs in the Drosophila melanogaster female germline. *Elife* **10** (2021).
4. Courret, C., Wei, X. & Larracuente, A.M. New perspectives on the causes and consequences of male meiotic drive. *Curr Opin Genet Dev* **83**, 102111 (2023).
5. Ridges, J.T. *et al.* Overdrive is essential for targeted sperm elimination by Segregation Distorter. *bioRxiv* (2024).
6. Mills, W.K., Lee, Y.C.G., Kochendoerfer, A.M., Dunleavy, E.M. & Karpen, G.H. RNA from a simple-tandem repeat is required for sperm maturation and male fertility in Drosophila melanogaster. *Elife* **8** (2019).

Dear Editor,

We are delighted to receive positive comments from reviewers. We have addressed all concerns raised by the reviewers. Please see our response (with reviewer comments in blue) as follows.

Hope these changes will suffice to make the manuscript suitable for Nature Communications.
Best wishes,
Yukiko

Reviewer #1 (Remarks to the Author):

I recommend publication of the revised manuscript, pending two minor clarifications detailed below.

I appreciate the authors' additional experiments using an independent AAGAG deletion line to assess sperm differentiation and HP2 localization. These data strengthen the conclusions regarding HP2 action on AAGAG DNA and the dose-dependent effect of AAGAG on sperm DNA condensation. While more experiments are needed to obtain definitive answers to whether HP2 functions as a direct activator and whether a formal checkpoint exists, I believe the authors did due diligence in their experiments and worded these interpretations as speculative.

Minor points:

Lines 112–113:
"However, given that HP2 expression decreased at the time of strongest AAGAG/CUCUU transcription in spermatocytes (Fig. 1g) …"
Is Fig. 1g the best figure for this sentence? Fig. S4a shows HP2 expression, but not Fig 1g.

We appreciate the reviewer catching this. We have changed the reference to Fig S4a.

Lines 188–190:
"Similar to the retention of H3K9me3, H3.3 histone was also retained in the defective spermatids in bam>HP2RNAi, whereas another histone mark, AcH4 …"
I could not find the AcH4 data referenced here.

We are very sorry for this mistake. It seems that somehow the older Figure was retained in the process of the previous round of revision. (For that matter, we had said that we revised the data presentation for Fig 4d, but the older version was retained. Now we replaced it with the promised-version of a dot plot graph for that figure, too).

Reviewer #2 (Remarks to the Author):

I appreciate the clarifications provided in the response and the paper.

Regarding the response about the original figure S8: I can appreciate the point about sample-to-sample signal variance but I would hope that this approach would work for detecting large differences, especially with a control. The published sequence-based estimates might not represent the flies in your hands, especially in repeat regions. I recommend going back to the original presentation and showing more images than the one, ideally from males, and then present the published genomic estimates alongside these images.

We have revised this supplementary figure accordingly. We added some ‘variations’ in FISH signal intensity (as sometimes the signal appears brighter than others). But we hope that the overall intensity difference is appreciable.

Reviewer #3 (Remarks to the Author):

The authors have addressed my previous concerns.

This reviewer had no additional concerns.
